# Supplementary material for: Analysis of MicroRNA Cargo in Circulating Extracellular Vesicles from HIV-Infected Individuals with Pulmonary Hypertension
Source: Cells. 2024 May 21;13(11):886. doi: 10.3390/cells13110886 (PMC11172129; doi:10.3390/cells13110886)
Supplement: Supplementary file 1 [file cells-13-00886-s001.zip › cells-2953607-supplementary.pdf]

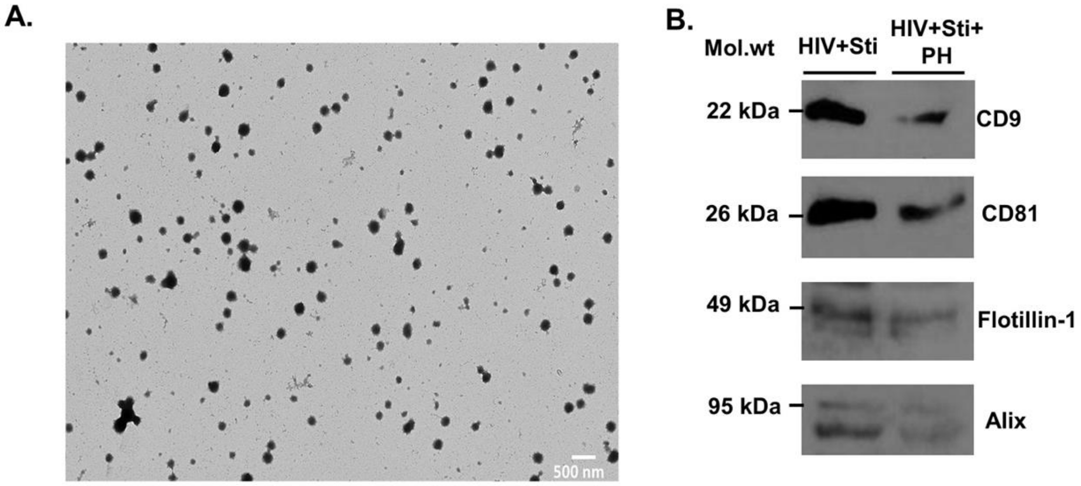

Supplementary Figure S1. Characterization of extracellular vesicles isolated from plasma of individuals with HIV+ Stim+ PH group and HIV+ Stim group. (A) Representative image of EVs based on Transmission Electron Microscopy (TEM). (B) Western blot analysis of EV markers. The same amount of total EV protein was loaded in both wells.

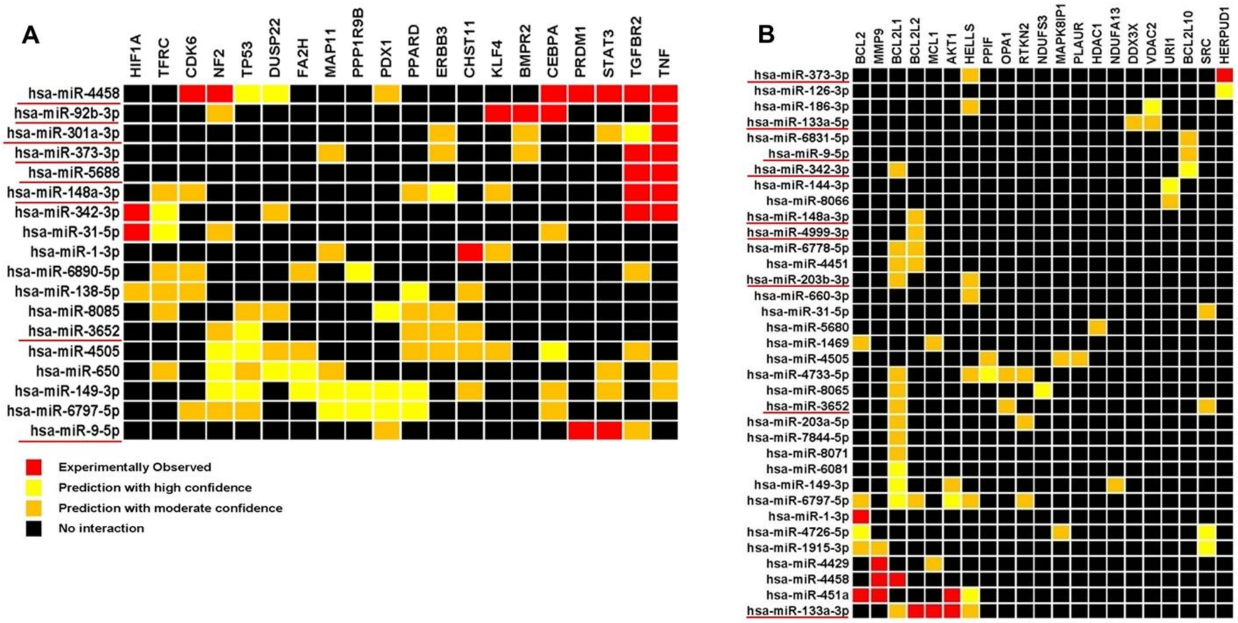

Supplementary Figure S2. Overrepresentation analysis of significantly up-regulated miRNAs depicted as heatmaps. A) The association matrix of the significantly up-regulated miRNAs and their target genes, in the “regulation of cell proliferation” pathway (GO0042127), depicted as a heatmap. B) The association matrix of the significantly up-regulated miRNAs and their target genes, in the “negative regulation of intrinsic apoptotic signaling” pathway (GO2001243), depicted as a heatmap.

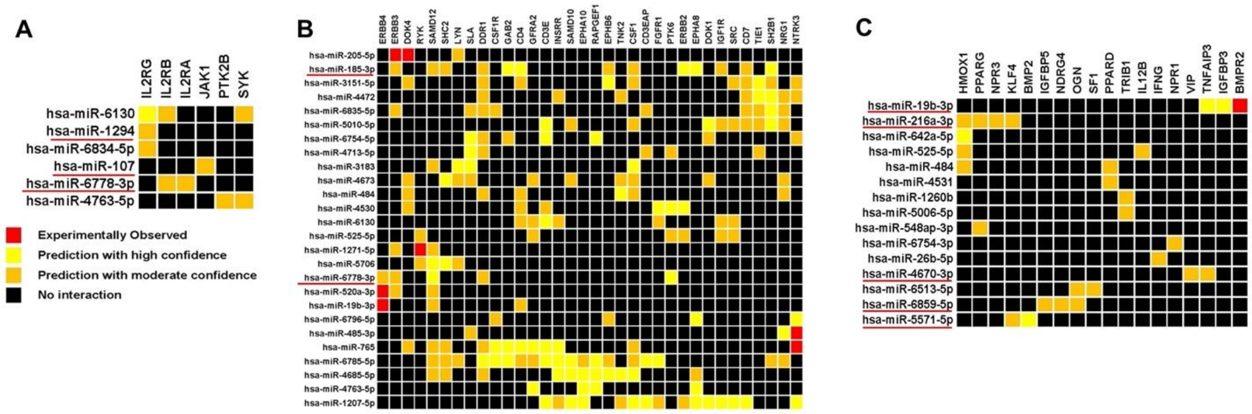

Supplementary Figure S3. Overrepresentation analysis of significantly down-regulated miRNAs**.** Depicted as heatmaps are the association matrix of the significantly down-regulated miRNAs and their target genes, in the A) "interleukin-2-mediated signaling" pathway (GO0038110), B) "transmembrane receptor protein tyrosine kinase signaling" pathway (GO0007169), and C) "negative regulation of smooth muscle cell proliferation" pathway (GO0048662).
